# The Impact of Oral Health on Taste Ability in Acutely Hospitalized Elderly

**DOI:** 10.1371/journal.pone.0036557

**Published:** 2012-05-03

**Authors:** Kirsten Solemdal, Leiv Sandvik, Tiril Willumsen, Morten Mowe, Thomas Hummel

**Affiliations:** 1 The Faculty of Dentistry, University of Oslo, Oslo, Norway; 2 Department of Medicine, Oslo University Hospital, University of Oslo, Oslo, Norway; 3 The Faculty of Medicine, University of Oslo, Oslo, Norway; 4 Smell and Taste Clinic, Department of Otorhinolaryngology, University of Dresden Medical School, Dresden, Germany; University of Toronto, Canada

## Abstract

**Objective:**

To investigate to what extent various oral health variables are associated with taste ability in acutely hospitalized elderly.

**Background:**

Impaired taste may contribute to weight loss in elderly. Many frail elderly have poor oral health characterized by caries, poor oral hygiene, and dry mouth. However, the possible influence of such factors on taste ability in acutely hospitalized elderly has not been investigated.

**Materials and Methods:**

The study was cross-sectional. A total of 174 (55 men) acutely hospitalized elderly, coming from their own homes and with adequate cognitive function, were included. Dental status, decayed teeth, oral bacteria, oral hygiene, dry mouth and tongue changes were recorded. Growth of oral bacteria was assessed with CRT® Bacteria Kit. Taste ability was evaluated with 16 taste strips impregnated with sweet, sour, salty and bitter taste solutions in 4 concentrations each. Correct identification was given score 1, and maximum total taste score was 16.

**Results:**

Mean age was 84 yrs. (range 70–103 yrs.). Total taste score was significantly and markedly reduced in patients with decayed teeth, poor oral hygiene, high growth of oral bacteria and dry mouth. Sweet and salty taste were particularly impaired in patients with dry mouth. Sour taste was impaired in patients with high growth of oral bacteria.

**Conclusion:**

This study shows that taste ability was reduced in acutely hospitalized elderly with caries activity, high growth of oral bacteria, poor oral hygiene, and dry mouth. Our findings indicate that good oral health is important for adequate gustatory function. Maintaining proper oral hygiene in hospitalized elderly should therefore get high priority among hospital staff.

## Introduction

Diminished taste perception and decreased ability to identify and discriminate basic taste qualities may deprive people of the pleasures of eating and of quality of life. Impaired taste may have serious consequences for sick elderly [1]. Poor appetite, weight loss and under-nutrition are frequently observed among elderly admitted to hospital [2]–[5]. In addition, acute disease often leads to depletion of proteins and essential nutrients which may further aggravate the patients' general health [6]. Taste loss may be one of several factors contributing to poor appetite, reduced dietary intake and weight loss in elderly patients. [7]–[10]. Adequate gustatory function is therefore important for these old patients in order to fight disease and regain a healthy constitution.

Reduced taste ability has been associated with gender [11], [12], increasing age [13], [14], diseases and drugs [15], [16]. Further, it has been claimed that some oral conditions, such as wearing dentures [17], dry mouth [18] and coated tongue [19], [20], may cause taste impairment. Many frail elderly have poor oral health, characterized by heavy plaque accumulation, mucosal inflammation, hypo-salivation and high caries activity [21]–[24]. However, as far as we know, only one study has examined the association between oral hygiene and taste ability before and after professional oral care [25]. Further, the possible influence of specific conditions such as caries and oral bacteria on taste ability has not been studied. To what extent compromised oral health in general may interfere with taste ability in acutely hospitalized elderly also remains to be investigated.

The aim of the present study was therefore to examine whether various oral health variables, such as caries, oral bacteria, oral hygiene, oral dryness and tongue changes may influence taste ability in such patients.

## Materials and Methods

### Ethics statement

The present cross-sectional study was approved by The National Committee for Medical and Health Research Ethics in Norway, and carried out at Oslo University Hospital, Aker, Norway between November 2009 and October 2010. Written informed consent was obtained from all participants.

### Study population

#### Inclusion criteria

Elderly at least 70 years old, living in their own homes prior to hospitalization for acute medical problems, and who met the inclusion criteria, were consecutively invited to participate. The patients were evaluated for participation at least 48 hours after hospital admission by two experienced physicians in geriatric medicine. The physicians were trained to evaluate patients for participation in clinical studies with similar guidelines and criteria. Evaluation of cognitive function was based on thorough interviews with the patients.

#### Exclusion criteria

Patients less than 70 years of age, patients with reduced cognitive function, patients coming from nursing homes, and patients with terminal diseases were not eligible for participation. Two hundred and thirty-four patients were asked to participate, and 200 accepted. Various reasons for non-attendance were as follows: “feeling too sick, too tired, just had my teeth checked, and I have only dentures, etc.”

Twenty-six patients did not have taste assessments. All twenty-six patients fulfilled the inclusion criteria. They had accepted to participate and had signed the letter of informed consent. However, when they were scheduled for taste testing, they were not available due to practical hospital logistics such as busy with laboratory tests, medical examinations or treatment, having visitors, eating, or dismissed from hospital. Drop out was not associated with the clinical condition of the patient, or any other underlying causes. They were not tested any time later. Thus, 174 patients were included in the study.

## Methods

### Examination of patients

The patients were instructed not to eat or drink one hour prior to the examination, because this could interfere with the taste testing and the registration of oral dryness. The oral examination was carried out at the bedside by a dentist, using two mirrors, a dental probe, and a head lamp. A predefined questionnaire was used to acquire information about age, education, and smoking as well as relevant clinical information. Information about the number of prescribed drugs was obtained from medical records in the hospital.

### Dental status

Dental status was registered according to WHO's Oral Health Surveys, Basic Methods 4th Edition [26]. The number of own teeth was noted. Teeth with crowns were counted as own teeth. A tooth was recorded as decayed if there was loss of tooth substance, and the tooth surface was soft on probing. In addition, “own teeth only”, “own teeth with dentures” or “dentures only” were recorded.

### Oral hygiene

Plaque on teeth and/or dentures (plaque score) and mucosal and/or gingival inflammation (mucosal score) were assessed with the Mucosal-Plaque Score (MPS) [27]–[32]. The index has been validated and tested for both intra - and inter- examiners agreement [27], [33]. The index has 4 graded scores for mucosal and/or gingival inflammation and 4 graded scores for the amount of plaque accumulation on teeth and/or dentures. Denture stomatitis, ulcers and decubitus are included in the mucosal score. The recordings for the mucosal score (MS) were as follows; (1) normal appearance of gingiva and oral mucosa, (2) mild inflammation, (3) moderate inflammation and (4) severe inflammation. The plaque score (PS) on teeth and/or dentures were as follows; (1) no easily visible plaque, (2) small amounts of hardly visible plaque, (3) moderate amounts of plaque, and (4) abundant amounts of confluent plaque. The MPS is the sum of mucosal score and plaque score, and the score range is from 2 to 8. Scores between 2 and 4 describe good or acceptable oral hygiene. MPS≥5 reflects un-acceptable/poor oral hygiene, and this score has been selected by experienced clinicians in previous studies according to the severity of the recorded oral hygiene status [27], [28], [31]. A criteria catalog with photos, showing examples of all the various conditions according to the graded scores, was presented for visual calibration [34].

### Oral dryness

To express oral dryness, three different variables were selected. These variables were the “mirror test” [35], registration of dry tongue and measurements of stimulated whole saliva. To assess dry mouth with the mirror test, the back of a dental mirror was moved across the inside of the buccal mucosa immediately after opening the mouth when starting the oral inspection. If the dental mirror was sticking to the mucosa, friction was noted. Dry tongue was recorded if the tongue was completely devoid of moisture. Assessment of stimulated whole saliva was done with the “chewing and spitting method” [36] The patients were asked to chew on paraffin wax for approximately 1 min. to soften the wax. After emptying the mouth of saliva, the timer was started. The patients were instructed to chew vigorously on the paraffin wax for 3 minutes while spitting into pre-weighted plastic container, whenever needed. The amount of saliva collected during paraffin wax chewing, was immediately weighted on a Precisa 2200C electronic scale (Precisa Gravimetrics AG, 8953 Dietikon, Switzerland), which was calibrated daily. Hypo-salivation was defined as stimulated whole saliva ≤0.6 g/min.

### Bacterial growth

The amount of *Streptococcus Mutans* and *Lactobacilli* in stimulated whole saliva were assessed with the CRT® Bacteria Kit (Ivoclar Vivadent AG, FL-9494 Schaan, Lichtenstein) [37]. The collected, stimulated whole saliva was dripped onto the growth medium and incubated for 48 hours at 37 C °. The CRT bacteria count was expressed as low colony forming units (CFU<10^5^/ml saliva) and high colony forming units (CFU≥10 ^5^/ml saliva). Some patients had severe problems with chewing paraffin wax due to either dry mouth or poorly fitted dentures. Thus, 158 patients had stimulated whole saliva collected, and 153 patients had valid assessments of oral bacteria.

### Tongue changes

Coated tongue was defined as a thick layer of plaque on the anterior dorsum of the tongue to be scraped off [38]. The tiny layer of white coating from normal shedding of filiform papillae was not recorded as tongue coating. The tongue was classified as atrophic if at least 50% of the tongue was devoid of papillae. In addition to visual inspection of the tongue, photos were taken for verification of the clinical diagnosis.

### Taste ability testing

Whole mouth gustatory function was assessed with the “taste strips” method [39]. The method has been validated and calibrated against the well-established “three-drop taste test” and shown to give a significant correlation with the “three-drop taste test” [40]. The taste strips were prefabricated and impregnated with sweet, sour, salty, and bitter taste solutions in four different concentrations each. The concentrations were: *Sweet taste*; 0.05, 0.1, 0.2 and 0.4 g/ml sucrose, *sour taste*; 0.05, 0.09, 0.165 and 0.3 g/ml citric acid, *salty taste*; 0.016, 0.04, 0.1 and 0.25 g/ml NaCl and *bitter taste*; 0.0004, 0.0009, 0.0024, 0.006 g/ml quinine–HCl. The strips were given to the participants according to a predefined procedure, starting with the weakest concentration and ending with the strongest. Both participant and examiner were blinded as to which taste quality or taste concentration given to the participant. The individual taste strip was placed in the middle of the anterior region of the tongue. The patients were allowed to suck on the strip for maximum twenty seconds. A poster with the words; *sweet*, *sour*, *salty*, *bitter*, was placed on the table in front of the patients who had to decide on one of the taste qualities without delay. Before starting, and in between every taste strip, the patients were asked to rinse with water to cleanse the mouth. The patients confirmed that the former taste had disappeared before the next taste strip was placed on the tongue. Correct identification was given score 1 and incorrect identification, score 0. The correct scores were summarized, and maximum total taste score was 16. Each individual taste quality was also given a sum score, ranging from 0 to 4.

### Sample size determination

Information about variability in taste ability, where taste was measured with the same “taste strip” method as in our study, was available from a pilot study, where the standard deviation of the total taste score was 2.5. We thus assumed that the corresponding standard deviation would be 2.5 in the planned study. We considered differences ≥1 in mean total taste score between two groups to be of clinical interest. It was shown that with 174 patients included in our study, we had at least 80% test power to detect a mean difference in total taste score of at least 1 between two subgroups, provided that the smallest subgroup contained at least 30 patients. Thus, our study appeared to have acceptable test power.

### Statistics

When comparing continuous variables in two groups, a two-sided independent samples t-test was used as long as the distribution of the variables was sufficiently close to normal distribution. If not, a two-sided Mann-Whitney test was used. Spearman's rho was applied to test associations between two continuous variables. Multivariate linear regression analysis was used to adjust for gender. The level of significance was set to 5%. PASW statistics, version18.0 (SPSS INC. Chicago, IL 60611, USA) was applied for statistical analysis. Quality assurance of data transfer from paper records was carried out by monitoring every 10th record.

## Results

### Demographic data and clinical variables

The mean (SD) age of 174 patients (n = 55 men) with valid taste scores was 83.5±6.1 years. Age ranged from 70 to 103 years. The prevalence of current smoking was 12% and the proportion of patients with education level of at least 12 years was 24%. The mean (SD) number of daily medications was 6.9±3.3. The mean (SD) number of natural teeth was 14.5±10.0 (n = 174). Mean (SD) plaque score was 2.3±0.8, mean (SD) mucosal score was 1.9±0.6 and mean (SD) MPS was 4.2±1.1. Mean (SD) stimulated whole saliva (158 patients) was 0.9±0.7 g/min. Stimulated whole saliva was significantly associated with the mirror test and dry tongue (r = −0.38, p<0.001, r = −0.41, p<0.001), respectively.

Table 1 presents various oral health variables such as dental status, oral hygiene, and oral bacteria. Further, table 1 shows the proportion of patients with oral dryness and tongue changes (coated tongue and atrophic tongue).

**Table 1:** The prevalence of various oral health variables in 174 hospitalized elderly with data presented as numbers (n) and proportions (%).

Dental status	Own teeth only, n (%)	98 (56.3)
	Own teeth with dentures, n (%)	44 (25.3)
	Dentures only, n (%)	32 (18.4)
	1Decayed teeth, n (%)	57 (40.8)
Oral hygiene status	2MPS≥5, n (%)	65 (37.3)
Oral bacterial growth	3Lactobacilli≥10^5^ CFU/ml saliva, n (%)	129 (84.3)
	3Streptococcus ≥10^5^ CFU/ml saliva, n (%)	123 (80.4)
Oral dryness	Dry tongue, n (%)	22 (12.6)
	Friction with mirror test, n (%)	26 (14.9)
	4Hyposalivation, n (%)	69 (43.7)
Coated tongue, n (%)		44 (25.3)
Atrophic tongue, n (%)		48 (27.6)

1 Prevalence of participants with number of teeth >0 (n = 142).

2 MPS (Mucosal –Plaque score) is the sum of the Mucosal score and the Plaque score with a sum score from 2 to 8. Un-acceptable/poor oral hygiene is defined as MPS≥5.

3 Number of patients with valid bacteria test was 153 patients.

4 Hyposalivation is defined as stimulated whole saliva ≤0.6 g/ml. The number of patients with collected stimulated whole saliva was 158 patients.

### Oral health variables and total taste score

Table 2 shows that there was no significant difference in total taste score between patients with “dentures only” and “patients with own teeth only”. Patients with un-acceptable oral hygiene (MPS≥5) had significantly lower total taste score than patients with acceptable oral hygiene (p = 0.009). Patients with high growth of *Streptococcus Mutans* and *Lactobacilli* had significantly lower total taste score than patients with low bacterial growth (p = 0.021 and p = 0.011), respectively. Patients with friction with the dental mirror and patients with dry tongue had significantly lower total taste score than patients without friction and normal moist tongue (p = 0.002 and p = 0.043), respectively. There was no significant difference in total taste score between patients with and without tongue coating or between patients with and without atrophic tongue.

**Table 2:** Associations between oral health variables and total taste score^1^ in 174 hospitalized elderly.

Oral health variables	n	Total taste score, mean (SD)	p- value ( p-values (adjusted for gender)
Dentures only	32	8.5 (2.4)	0.70
Own teeth only	98	8.7 (2.6)	
2 Acceptables oral hygiene, (MPS<5)	109	9.1 (2.4)	0.009*
Un-acceptable oral hygiene, (MPS≥5)	65	7.9(2.9)	(0.004*)
3Streptococcus M<10^5^ CFU/ml saliva	30	9.7(2.3)	0.021*
3Streptococcus M≥10^5^ CFU/ml saliva	123	8.5 (2.6)	(0.014*)
3Lactobacilli <10^5^ CFU/ml saliva	24	10.0 (2.5)	0.011*
3Lactobacilli ≥10^5^ CFU/ml saliva	129	8.5 (2.6)	(0.010*)
No friction with mirror test	148	8.9 (2.6)	0.002*
Friction with mirror test	26	7.2 (2.6)	(0.001*)
No dry tongue	152	8.8 (2.6)	0.043*
Dry tongue	22	7.6 (2.7)	(0.007*)
No coated tongue	130	8.9 (2.6)	0.09
Coated tongue	44	8.1( 2.9)	
No atrophic tongue	126	8.9 (2.6)	0.09
Atrophic tongue	48	8.1 (2.7)	

1 Total taste score is the sum of correct identifications of taste strips (maximum score = 16).

2 MPS is the sum score of Mucosal inflammation score and Plaque score. The score is from 2 to 8. Acceptable oral hygiene is defined as MPS<5, and unacceptable/poor oral hygiene is defined as MPS≥5.

3 The oral bacteria *Streptococcus Mutans* and *Lactobacilli* were assessed in 153 patients.

* p≤0.05.

In cases where significance was reached, p-values adjusted for gender are shown in parentheses below unadjusted p-values.

### Oral health variables associated with total taste score, sour and salty score

Table 3 shows that total taste score was reduced in patients with high number of decayed teeth (r = −0.22, p = 0.008), high plaque score (r = −0.16, p = 0.035), high mucosal score (r = −0.15, p = 0.048) as well as high mucosal-plaque sum score (r = −0.20, p = 0.010). Low sour sum score was associated with decayed teeth (r = −0.21, p = . 0.015) and high plaque score (r = −0.17, p = 0.027), but not with the other oral health variables listed. Low sum score for “salty” was associated with increasing mucosal inflammation score (r = −0.20, p = 0.009) and mucosal–plaque sum score (r = −0.15, p = 0.045). Further, the salty sum score was positively associated with stimulated whole saliva (r = 0.18, p = 0.023). Sweet and bitter sum scores were not significantly associated with any of the oral health variables presented in table 3 (data not shown).

**Table 3:** Total taste score, sour and salty sum scores associated with different oral health variables in 174 hospitalized elderly.

Oral health variables	Total taste score		Sour sum score		Salty sum score	
	r	p-value (adjusted)	r	p-value (adjusted)	r	p-value (adjusted)
1Decayed teeth	−0.22	0.008* (0.001*)	−0.21	0.015* (0.10)	−0.13	0.14
2Plaque score	−0.16	0.035* (0.032*)	−0.17	0.027* (0.031*)	−0.04	0.59
3Mucosal score	−0.15	0.048* (0.022*)	−0.012	0.89	−0.20	0.009* (0.009*)
4Mucosal-Plaque sum score	−0.20	0.010* (0.004*)	−0.13	0.09	−0.15	0.045* (0.037*)
^5^Stimulated saliva g/min	0.12	0.15	−0.10	0.23	0.18	0.023* (0.026*)

1 The number of dentate patients were 142.

2 Plaque score is defined as plaque on teeth and/or dentures, (graded from 1–4).

3 Mucosal score is defined as mucosal and/or gingival inflammation, (graded from1–4).

4 Mucosal-Plaque Score (MPS) is the sum of Plaque score and Mucosal score with sum score from 2 to 8.

Stimulated whole saliva was collected in 158 patients.

* p≤0.05.

In those cases where significance was reached, the p-values adjusted for gender are shown in parentheses below the unadjusted p-values.

Sweet and bitter sum scores were not significantly associated with any of these variables, and are therefore not shown in the table.

### Dichotomous oral health variables and taste quality

Table 4 shows the influence of various dichotomous oral health variables on the basic taste qualities sweet, sour and salty. Sweet sum score was significantly lower in patients with friction with the dental mirror, dry tongue and atrophic tongue (p = 0.007, p = 0.001, and p = 0.009), respectively. Sour sum score was significantly lower in patients with high growth of Lactobacilli (p = 0.001). Salty sum score was significantly lower in patients with friction with the dental mirror and dry tongue (p = 0.009 and p = 0.030) respectively, both indicative of dry mouth. The possible influence of these oral health variables on bitter sum score is not presented in the table due to lack of significant differences.

**Table 4:** Impact of various oral health variables on sum scores of sweet, sour and salty taste qualities in mean (SD) in 174 hospitalized elderly.

Oral health variables	Sweet sum score p-value (adjusted)	Sour sum score p-value (adjusted)	Salty sum core p-value (adjusted)
1Streptococcus M<10^5^ CFU ml/saliva	3.4 (0.8)	1.2 (0.9)	2.3 (1.2)
1Streptococcus M≥10^5^ CFU ml/saliva	3.0 (1.1)	0.9 (0.8)	2.0 (1.2)
	p = 0.13	p = 0.10	p = 0.26
2Lactobacilli<10^5^ CFUml/saliva	3.4 (0.8)	1.5 (0.8)	2.0 (1.3)
2Lactobacilli≥10^5^ CFUml/saliva	3.0 (1.1)	0.9 (0.8)	2.1 (1.1)
	p = 0.17	p = 0.001* (0.001*)	p = 0.84
No friction with mirror test	3.2 (1.0)	1.0 (0.9)	2.1 (1.1)
Friction with mirror test	2.4 (1.3)	0.9 (0.8)	1.5 (1.1)
	p = 0.007* (0.001*)	p = 0.78	p = 0.009* (0.009*)
No dry tongue	3.2 (1.0)	0.9 (0.8)	2.1 (1.2)
Dry tongue	2.2 (1.3)	1.1(0.9)	1.6(1.0)
	p = 0.001* (<0.001*)	p = 0.31	p = 0.030* (0.027*)
No atrophic tongue	3.2 (1.0)	1.0 (0.9)	2.1 (1.1)
Atrophic tongue	2.7 (1.2)	1.0 (0.8)	1.9 (1.2)
	p = 0.009* (0.002*)	p = 0.74	p = 0.36

* p≤0.05.

Significant p-values are adjusted for gender and shown in parentheses. None of the oral health variables had significant impact on bitter taste quality, and the results are therefore not presented in the table.

1,2 *Streptococcus Mutans* and *Lactobacilli* were assessed in 153 patients.

### Confounding factors

Potential confounding factors in this study might be age, gender, smoking, education, and number of medications. All were tested against the outcome variables. However, only gender was significantly associated with the taste scores. Thus, all the significant findings were adjusted for gender only. The majority of the oral health variables with significant associations with total taste score, sweet, sour and salty taste scores remained significant after adjusting for gender (table 2 and 3). However, the association between sour taste and decayed teeth was no longer significant after adjusting for gender.

## Discussion

In the present study we show that taste ability is significantly impaired in acutely hospitalized elderly with decayed teeth, high growth of oral bacteria associated with caries, poor oral hygiene and dry mouth. The majority of these findings remained significant after adjusting for gender.

Caries is associated with *Lactobacillus*, *Streptococcus Mutans* and poor oral hygiene [41], [42]. It has been suggested that taste loss associated with poor oral health could be due to toxins and inflammatory products produced by the oral bacteria [43]. In our study, sour taste was particularly impaired in patients with high lactobacilli growth. These oral bacteria proliferate in an acidic environment, and they also produce acid themselves. Although the mechanisms involved in taste transduction are rather complex [44], [45], an explanation could be that the acid produced by the bacteria may cause adaption in sour taste perception, and thus increasing the taste threshold for sour. Further, we found that poor oral hygiene was associated with reduced total taste score and salty taste. This is in agreement with Langan et al. [25], concluding, in a small study with 15 participants, that professional oral hygiene improved taste acuity for salty taste as well as sweet taste.

In our study, no difference in taste ability was found between patients with and without tongue coating. This is in contrast to a number of studies, e.g., Quirynen et al. [46], Ohno et al. [19], and Kostka et al. [20], reporting that taste sensitivity improved after tongue cleaning. However, their study design and methods were quite different from those in our study. We examined taste ability in patients with and without coated tongue, while they tested taste sensitivity before and after removal of tongue coating with tongue brushing.

Hospitalized elderly frequently use a high number of drugs daily, which may induce xerostomia and hypo-salivation [47]. Saliva is essential for bringing food particles and taste stimuli to the taste buds in the oral cavity [48]. Reduced saliva flow is reported to be associated with taste loss [49], [50]. In our study in acutely hospitalized elderly, dry mouth was associated with impaired taste. This is in accordance with both Kamel et al. [18] and Weiffenbach et al. [51], reporting that patients with Sjøgren's syndrome, which is characterized by oral dryness, had reduced taste sensitivity compared with controls.

Some studies claim that people with dentures have higher taste threshold [17], [52] and also elevated retronasal flavour threshold [53], than people without dentures. In our study there was no difference in taste scores between patients with full dentures and patients with natural dentition. Why our finding is not in line with those other studies is not known, but could be due to different methods used.

In our study, atrophic tongue was associated with reduced sweet score. It remains to be explained why atrophy of the tongue papillae affected the sweet taste quality more than the other taste qualities.

### Limitations and strengths

Our study has some limitations. The “mirror test” is a crude clinical method for estimation of buccal dryness. This test has not been properly validated. However, it has been used as a clinical reference when more sophisticated devices have been tested for possible use in assessing buccal dryness [35]. Furthermore, the registrations of caries were not performed under optimal conditions, and no x-rays were taken to support the findings. This could cause underestimation of caries activity in these patients. Still we do not believe that this will change the main conclusions of our study. When assessing atrophic tongue based on visual inspection, there is always a risk of either over or under-estimations. However, the prevalence of atrophic tongue in our study was similar to a previous study at the same hospital ward [54]. Although the study took place in a difficult setting, we managed to collect substantial information about several oral health parameters and their associations with gustatory function in a relatively large number of very old and severely ill acutely hospitalized elderly. This is an important strength of our study.

### Conclusion

The present study shows that taste ability was significantly reduced in acutely hospitalized elderly with caries activity, high growth of oral bacteria, poor oral hygiene, and dry mouth. Our findings suggest that good oral health is important for adequate gustatory function in such patients. Maintaining proper oral hygiene in hospitalized elderly should therefore get high priority among hospital staff. Further, healthy oral conditions contributing to better taste perception, may stimulate appetite and enhance caloric intake. This may help to prevent nutritional deficiency in hospitalized elderly and improve the patients' general health and quality of life.
